# Molecular Mechanism of Aflatoxin-Induced Hepatocellular Carcinoma Derived from a Bioinformatics Analysis

**DOI:** 10.3390/toxins12030203

**Published:** 2020-03-23

**Authors:** Peirong Cai, Hao Zheng, Jinjin She, Nannan Feng, Hui Zou, Jianhong Gu, Yan Yuan, Xuezhong Liu, Zongping Liu, Jianchun Bian

**Affiliations:** 1 College of Veterinary Medicine, Yangzhou University, Yangzhou 225009, China; MX120170756@yzu.edu.cn (P.C.); yuanyan@yzu.edu.cn (Y.Y.);; 2 Jiangsu Co-innovation Center for Prevention and Control of Important Animal Infectious Diseases and Zoonoses, Yangzhou 225009, China; 3 Joint International Research Laboratory of Agriculture and Agri-Product Safety of the Ministry of Education of China, Yangzhou University, Yangzhou 225009, China

**Keywords:** aflatoxin, hepatocellular carcinoma (HCC), bioinformatics analysis, HRG, PCK2

## Abstract

Exposure to aflatoxin is considered to be one of the causes of hepatocellular carcinoma (HCC). With the development of bioinformation, we sought to reveal the occurrence and development of aflatoxin-induced HCC through data research. We identified differentially expressed genes (DEGs) of datasets GSE127791 (Aflatoxin-treated pluripotent stem cell derived human hepatocytes vs. controls) and GSE64041 (liver carcinoma with unknown cause vs. non-cancerous tissue) by GEO2R to find the common DEGs. Gene ontology (GO) and KEGG path enrichment analysis were used to annotate the function of DEGs. Hub genes were screened from identified DEGs by protein-protein interaction (PPI) network analysis. The prognostic value of hub genes in cancer databases were evaluated. We obtained 132 common DEGs and 11 hub genes. According to cluster analysis and protein co-expression networks, we screened out the key genes, histidine-rich glycoprotein (HRG) and phosphoenolpyruvate carboxykinase 2 (PCK2). Oncomine database and survival curve analysis showed that the decline in HRG and PCK2 expression in the development of HCC indicated poor prognosis. We speculated that the decreased expression of HRG and PCK2 after aflatoxin exposure to hepatocyte may be related to aflatoxin induced hepatocyte injury and carcinogenesis. In addition, the decreased expression of HRG and PCK2 in the occurrence and development of HCC suggests a poor prognosis of HCC.

## 1. Introduction

HCC (hepatocellular carcinoma) is one of the four major cancers found in the world with more than 700,000 new cases confirmed, which also accounts for more than 600,000 global deaths annually [1]. HCC is the main form of liver cancer which is the second most common cancer-related cause of death worldwide [2]. HCC accounts for 9.2% of all new diagnosed cancers, and nearly 90% of all primary HCCs, which is also recognized as one of the deadliest cancers [3]. In 2018, the number of new cases of HCC in the United States was estimated at 42,220 (30,610 males and 11,610 females) [4]. The death rate is estimated to be 30,200 (20,540 males and 9660 females) [5]. This number is somewhat depressing and has caused quite serious social and economic losses. Further research is needed to identify new therapeutic targets to fight HCC. 

About 60% of the world’s grains are contaminated with mycotoxins [6]. Toxin exposure, such as aflatoxin, as well as hepatic viral infections and the state of chronic hepatitis are linked to the development of HCC [7]. Aflatoxin, as a metabolite of mildew in the environment, is found to widely exist in nature. Its potential for provoking harm is self-evident [8]. There are approximately 18 identified aflatoxins; however, those of major concerns include AFB 1, AFB 2, AFG 1, AFG 2, AFM 1 and AFM 2 [9]. It is well known that aflatoxin exposure to hepatocyte can form DNA adducts and induce the mutation site of the tumor suppressor gene p53, which supports current thinking that aflatoxin is one of the possible factors of HCC [10]. AFB1 is the most toxic carcinogen, and as early as 1993 the International Agency for Research on Cancer (IARC) classified AFB1 as a class I human carcinogen [11]. 

Aflatoxin is mainly metabolized in the liver [12,13]. Studies demonstrate that AFB1 metabolites bind DNA via alkylation of bases that cause cell cycle alterations and the mutation of the tumor suppressor gene p53 [14]. However, the mechanism responsible for carcinogenesis is very complex. Although researchers have explained that a p53 mutation is a possible mechanism accounting for aflatoxin-induced HCC, there is no direct experimental evidence demonstrating that the p53 mutation induces HCC. What we have learned so far is that the occurrence of HCC is related to many factors, such as hepatitis B, hepatitis C, alcoholic and non-alcoholic fatty liver, mycotoxins, etc. [15]. Since the incidence of HCC continues to rise, this indicates a role of environment which again means that HCC has not been effectively controlled. In order to control aflatoxin-induced HCC, we need to further study the carcinogenic mechanism of aflatoxin.

In the era of high-tech development, experimental analysis with the assistance of big data analysis represent a new and practical means for the interrogative and systematic analysis of very large sets of targeted data. Over the past decade, a large number of molecular biological information-rich databases and meta-databases have been published. Genomic sequencing, protein arrays, and microarray analyses all represent effective means for high-throughput sequencing and are collectively uploaded to public databases, which include the Gene Expression Omnibus (GEO) and Oncomine databases that are employed to understand molecular changes in pathological tissues [16]. 

At present, some researchers have explored the mechanism of cancer through bioinformatics analysis [17,18]. To the best of our knowledge, there is no bioinformatics analysis that has associated HCC development with aflatoxin. To better understand the mechanism of aflatoxin-induced HCC, combined with some open-sourced databases, public microarray data regarding aflatoxin-treated hepatocytes vs. controls, and liver carcinoma vs. non-cancerous tissue were downloaded and analyzed using bioinformatics methods. We explored and analyzed differentially expressed genes (DEGs) by systematic bioinformatics analysis. 

Our overarching aim was to explore the induction mechanism of aflatoxin-mediated HCC using large dataset analyses and to provide the discovery of potential biomarkers that might have utility in the diagnosis and development of novel therapeutic drugs.

## 2. Results

### 2.1. Screening and Identification of Differentially Expressed Genes (DEGs)

**Figure 1:**
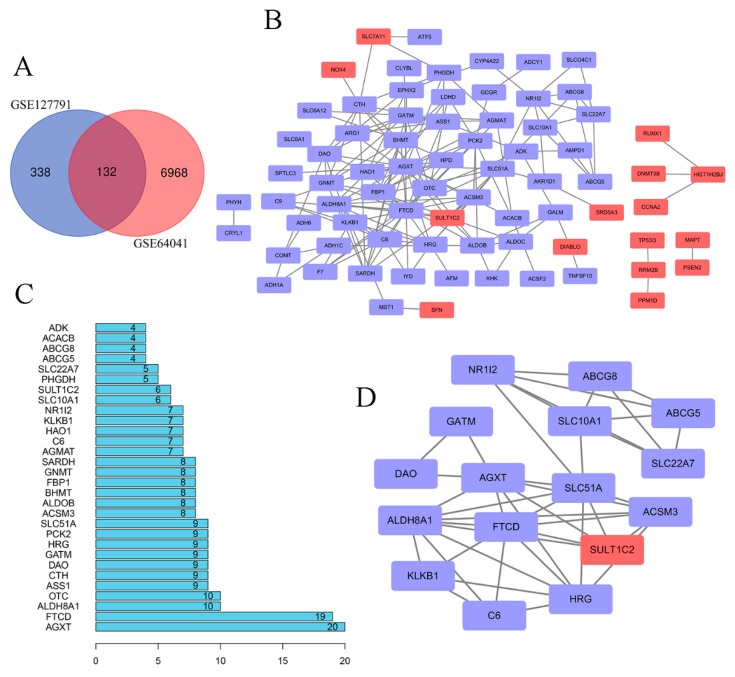
Screening of differentially expressed genes (DEGs) and analysis of the protein-protein interaction (PPI) network. (**A**) The common DEGs were screened by the intersection of GSE127791 and GSE64041. (**B**) Demonstration of PPI in DEGs by Cytoscape. (**C**) Analysis of DEGs based on PPI analysis results.(**D**) The most important module was obtained from a PPI network with 16 nodes and 42 edges. Upregulated genes are marked in red; downregulated genes are marked in light purple.

After standardization of the microarray results of the datasets, 132 DEGs were obtained by drawing the Venn diagrams with online tools (Figure 1A). The list of DEGs were shown in Table S1 in supplementary materials. By assessing potential protein interactions, possible mechanisms for disease progression can be identified. The analysis of the protein-protein interaction (PPI) network using String tools was used, in which we hid disconnected nodes in the network since our aim is to understand the relationship between gene interactions (Figure 1B). By differential analysis, 15 upregulated genes and 60 downregulated genes were screened out. The analysis of PPI was shown in Table S2 in supplementary materials. Next, the first 30 DEGs, which was shown in Table S3 in supplementary materials, were sorted by the R statistical language tool to list histograms of key genes (Figure 1C). The most significant module was obtained from the PPI network with 16 nodes and 42 edges (Figure 1D).

### 2.2. Enrichment Analysis of GO and KEGG of Differentially Expressed Genes (DEGs) 

**Figure 2:**
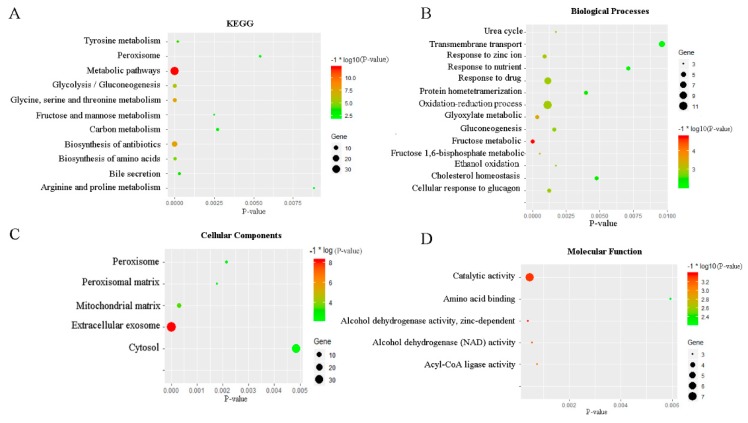
Pathway enrichment analysis. (**A**) Enrichment analysis of KEGG pathway. The Y-axis represents the category of KEGG, and the X-axis represents -log10 (P-value). (**B**) Biological process in enrichment analysis of GO pathway. The Y-axis represents the category of biological process, and the X-axis represents -log10 (P-value). (**C**) Cellular components in enrichment analysis of GO pathway. The Y-axis represents the category of cellular components, and the X-axis represents -log10 (P value). (**D**) Molecular functions in enrichment analysis of GO pathway. The Y-axis represents the category of molecular functions, and the X-axis represents -log10 (P value).

According to DAVID’s functional and pathway enrichment analysis, we selected P-value ≤ 0.01 as the screening cut-off criterion (Figure 2). The detailed information of the enrichment analysis was shown in Table S4 in supplementary materials. The results of the KEGG enrichment analysis showed that DEGs were mainly enriched in the processes of biosynthesis and metabolism (Figure 2A). It is mainly summarized as amino acid metabolism and carbohydrate metabolism, and as antibiotic and amino acid synthesis.

GO term analysis showed that the fructose metabolic process was the most significant enrichment in the biological processes, followed by the glyoxylate metabolic process, followed by the fructose 1,6-bisphosphate metabolic process (Figure 2B). The most significantly enriched gene sets in the cellular components were mainly aligned to the extracellular exosome and mitochondrial matrix (Figure 2C). Changes in molecular function were mainly enriched in the activities of acyl-CoA ligase, alcohol dehydrogenase (NAD), and other catalytic activities (Figure 2D).

### 2.3. Hierarchical Clustering of Hub Genes

**Figure 3:**
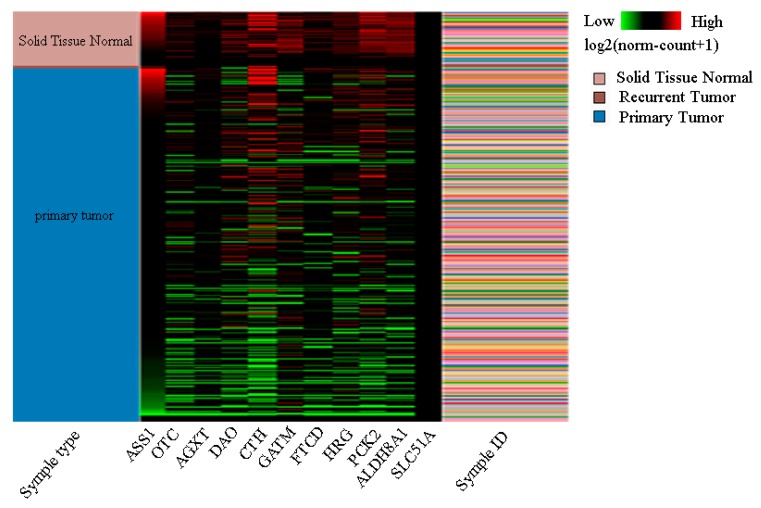
Using University of California Santa Cruz (UCSC) Xena to construct hierarchical clustering of hub genes. In the gene expression module, red represents upregulated, green represents downregulated, and black represents no change. In the symple ID module, different colors represent different samples.

We selected a degree ≥ 9 as the hub genes. Hierarchical clustering of hub genes was performed using the UCSC Cancer Genomics online tool. In the heatplot, according to the tumor tissues and normal tissues in the symple type module, we can observe the expression of hub genes in tumor tissues and normal tissues in the middle module, in which red represents upregulated, green represents downregulated, and black represents no changes. In the symple ID module, different colors represent different samples, which means that the gene expression in the middle module comes from different samples. As shown as Figure 3, SLC51A was not recorded in the UCSC database; thus, we ceased further analysis of this gene because it was not an important gene in the context of HCC. In addition, due to the fact that the extent of upregulation in the genes for CTH and ASS1 was almost the same as those that were downregulated, the remainder of the genes were essentially downregulated in HCC. This approach can be used to differentiate HCC from normal tissues. 

### 2.4. HRG and PCK2 Were Key Genes in the Hub Gene Dataset

**Figure 4:**
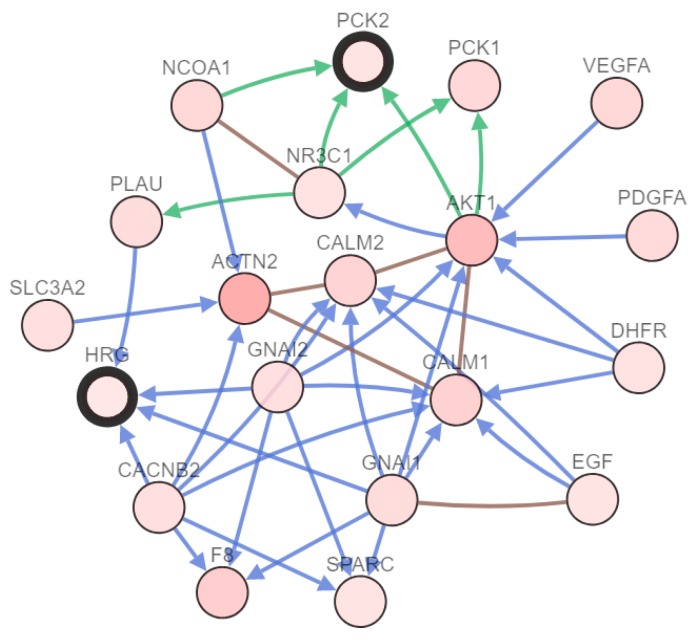
Using cBioportal online tool to construct a network of hub genes and their co-expression genes. The black outline of the thick line represents the central gene, while the light black represents the co-expression gene.

The hub genes and their co-expressed genes were analyzed by employing the cBioPortal online database. As shown as Figure 4, the nodes of the blackbody contour represent the hub genes. In other words, cBioportal analysis showed that both phosphoenolpyruvate carboxykinase 2 (PCK2) and histidine-rich glycoprotein (HRG) were key genes in the hub gene dataset.

### 2.5. Decreased Expression of HRG and PCK2 in HCC

**Figure 5:**
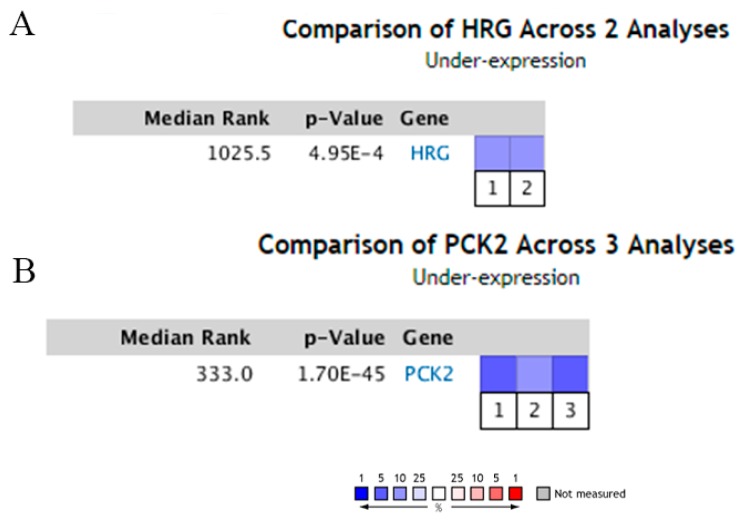
Oncomine analysis of the expression of normal tissues vs. HCC. The threshold is as follows: P-value = 0.01, fold change = 2, gene rank = top 10%.

In order to validate the clustering analysis results, we used the oncomine online database to search and analyze. The filtering conditions were gene: HRG, type: cancer vs. normal analysis, cancer type: HCC. The expression of HRG in HCC was significantly lower than that in the normal control group (P < 0.01) (Figure 5A). The expression level of PCK2 in HCC tissue was also significantly lower than that in the normal control group (*p* < 0.01) (Figure 5B). 

### 2.6. Survival Rate Analysis of HRG and PCK2 Expression of HCC Patients

**Figure 6:**
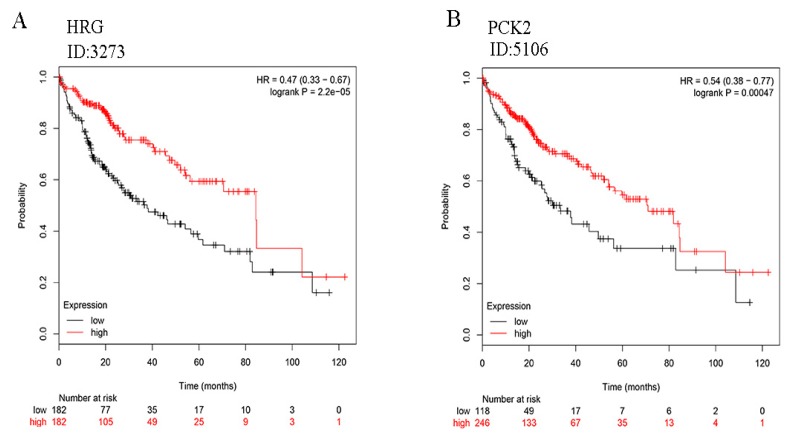
The overall survival analysis of Histidine-rich glycoprotein (HRG) and phosphoenolpyruvate carboxykinase 2 (PCK2) in HCC using the Kaplan-Meier curve method. An alpha value of P ≤ 0.01 is considered a statistically significantly difference. (**A**) The survival curve of HRG. (**B**) The survival curve of PCK2. The red curve indicates an increase in gene expression and the light black curve indicates decreased gene expression.

To further study the prognostic significance of HRG and PCK2 expression in patients with HCC, the combined Kaplan–Meier curve analysis revealed that the overall survival rate of patients with HCC was poor and exhibited decreased mRNA expression of both HRG and PCK2 in HCC (Figure 6A,B). These results suggest that decreased HRG and PCK2 expression in HCC might predict poor prognosis. 

## 3. Discussion

As the analysis of biological systems and datasets becomes increasingly complex, biomedical research is also increasingly dependent on knowledge stored in computable form. Microarray analysis or high-throughput sequencing can quickly identify DEGs in cancer specimens. These technologies have broad application and prospects in clinical research, including molecular classification, targeted drug discovery and survival prediction [19,20]. The occurrence of HCC involves a variety of inducements; the cases found in the human body are also more complicated due to the differences of various environmental and dietary factors. In these cases, it is not clear which factor causes the occurrence of HCC, which is often difficult to be determined clinically. Aflatoxins are clearly defined as the first class of carcinogens, especially HCC. However, due to human ethics and other factors, we cannot conduct toxicology experiments in the human body at will, which also limits the study of toxicology in humans. In the GSE64041 dataset, it is not clear what causes patients to have a HCC, but our purpose is to explore the mechanism of HCC induced by aflatoxin through the connection between these unknown causes of HCC and aflatoxin. Through bioinformatics analysis, aflatoxin exposure to human hepatocytes is related to human HCC. Taking normal hepatocytes as the bridge of connection can prompt us to understand the changes of key genes of aflatoxin-induced hepatocarcinogenesis in a new way. This new way of analysis is also the innovation of our article. Many studies have explored the carcinogenic mechanism of aflatoxin, but many of them lack clinical evidence. At present, the carcinogenic toxicity of aflatoxin has not been studied in the direction of large data mining approaches. From the perspective of bioinformatics, we look forward to providing meaningful research directions for aflatoxin research.

In our work, we have focused on the analysis of 132 DEGs that were screened from two datasets (Figure 1). We found that the common ground between gene expression in aflatoxin-induced toxicity and cancer cells was mainly concentrated in cellular biosynthesis and metabolism, which can be obtained from KEGG analysis. Of course, the combined observation of GO term’s results can markedly affect the function of glucose metabolism. When we distinguished the first eleven important genes by adopting a limitation of the degree, we found that SLC51A did not significantly benefit from the next level of analysis provided by clustering analysis; thus, we excluded SLC51A. The remaining ten genes were analyzed by mapping the gene co-expression network by cBioportal, and we condensed the key genes once again, leaving only PCK2 and HRG.

**Figure 7:**
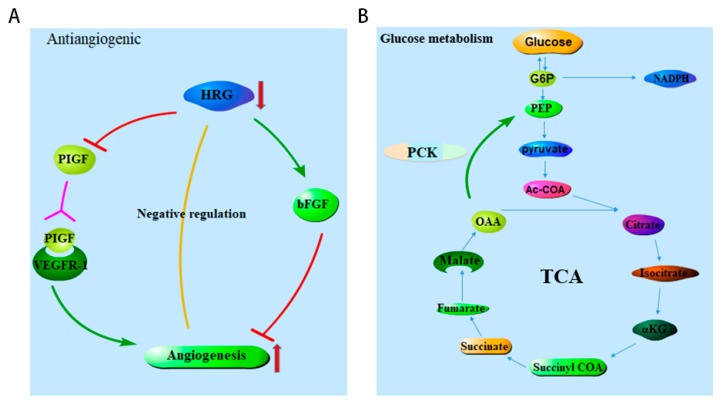
Physiological functions of HRG and PCK2 in normal cells. (**A**) HRG inhibits vascularization and tumor establishment. HRG, histidine-rich glycoprotein; PIGF, placental growth factor; VEGFR-1, Vascular endothelial growth factor receptor 1; bFGF, basic fibroblast growth factor; (**B**) PCK involved in gluconeogenesis. PCK, phosphoenolpyruvate carboxykinase; G6P, glucose 6-phosphate; PEP, phosphoenolpyruvate; OAA, oxaloacetate; α-KG, α-ketoglutarate.

Histidine-rich glycoprotein (HRG) is a plasma protein synthesized by the liver, which can regulate many important biological processes such as angiogenesis, pathogen clearance, cell adhesion, coagulation and so on. Among the most closely related to the occurrence of cancer is the regulatory effect of HRG on angiogenesis (Figure 7A). HRG can inhibit the expression of angiogenic placental growth factor (PlGF). According to relevant research, PlGF is overexpressed in many types of tumors and promotes angiogenesis by binding VEGFR-1 [21,22,23]. In other words, HRG can inhibit tumor angiogenesis. HRG has been shown to inhibit tumor growth and metastasis and does so by downregulating PlGF to induce macrophage polarization and normalization of blood vessels [24]. HRG promotes the polarization of hepatic macrophages to the M1 phenotype, which aggravates chronic liver injury and fibrosis [25] and restricts the growth of blood vessels [26]. In some studies, HRG deficient mice are protected from liver injury and liver fibrosis, which are two prerequisites for the occurrence of HCC in human liver diseases [27]. By contrast, HRG-deficient mice exhibit increased hemorrhagic duct formation without liver injury or inflammation, which may be a major pre-requisite in promoting the development of HCC [28]. In this current study, the survival analysis of HRG in HCC also showed that the decreased mRNA expression of HRG led to a poor prognosis in HCC, indicating the importance of HRG in HCC onset. Glycosylation is one of the most common post-translational modifications of proteins. In the study of HCC, it was found that the glycosylation site mutation of HRG affected the interaction between HRG and heparin sulfate (HS), which may be the key reason for the decrease of HRG in HCC [29]. Phosphoenolpyruvate carboxykinase 2 (PCK2), a mitochondrial subtype of phosphoenolpyruvate carboxy kinase (PEPCK), catalyzes the conversion of oxalic acid to phosphoenolpyruvate in the presence of guanosine triphosphate (Figure 7B). PEPCK can convert other precursors of lactate and citric acid cycle into glucose, which is a rate-limiting step in the metabolic pathway [30,31]. As we have learned, the metabolic regulation mechanism of cancer cells is complex and diverse in order to support their survival and growth under limited nutritional conditions [32]. Many cancer cells, such as thyroid cancer, bladder cancer, breast cancer and small cell lung cancer, can promote energy metabolism by upregulating PK2. [31,33,34]. However, PCK2 is a double-edged sword in terms of the occurrence and development of tumors. PCK2 plays an anti-cancer role in HCC and melanoma [35,36], while PCK2 also plays a carcinogenic role in both lung and breast cancer. In the study of HCC, increasing the expression of PCK can lead to energy crisis, truncated gluconeogenesis, TCA cycle rupture and a high ROS level, which can trigger the glucose-deprivation in HCC cells [36]. In the study of aflatoxin on mitochondria, it was found that aflatoxin could increase the concentration of the DNA adduct in mitochondria of mice liver, which was three to four times higher than that of nuclear DNA adduct and remained unchanged 24 hours later [37]. In the study of aflatoxin on chicken hepatocytes, it was found that aflatoxin can induce ROS production and lead to mitochondrial function damage [38]. We speculated that the damage of aflatoxin to mitochondria reduced the expression of PCK and accelerated the transformation of hepatocytes into a hepatoma. We speculated that the damage of aflatoxin to mitochondria reduced the expression of PCK, led to the disorder of gluconeogenesis, and accelerated the transformation of hepatocytes into liver cancer. However, the downregulation mechanism of PCK2 in HCC has not been reported yet, but researchers speculated that the downregulation of gluconeogenesis enzymes seems to be beneficial to the development of tumors in gluconeogenic tissues, which constitutes a part of metabolic recombination necessary to support tumor growth and proliferation [39]. The downregulation mechanism of PCK2 expression is also a potential direction in the study of HCC. The sequencing results of the GSE127791 dataset showed the negative regulation of aflatoxin on the expression of HRG and PCK2. However, further studies are needed to verify the role of HRG and PCK2 in the carcinogenesis of aflatoxin.

In the analysis of this study, we also found that downregulated PCK2 expression in the context of the onset of HCC can ultimately lead to a poor prognosis from this disease. We speculate that PCK2 might play an important role in aflatoxin-induced hepatocyte injury or transformation as well as the occurrence and development of HCC, which might also formally represent one of the key factors that influences the prognosis of HCC. However, further confirmatory studies are needed.

Until now, there are no additional detailed studies of the gene expression of HRG and PCK2 after hepatocytes exposure to aflatoxin. We believe that the decline in HRG and PCK2 that is induced by aflatoxin is closely related to the occurrence and development of HCC, as revealed by joint analysis of the available datasets.

The aim of this study was to identify the key genes that might be related to the occurrence or progression of aflatoxin-induced HCC. Through our data mining approaches, we focused on two genes, HRG and PCK2. These findings suggested that HRG and PCK2 may be potential targets for aflatoxin-HCC diagnosis and treatment. In the meantime, we cannot ignore the fact that the regulation of messenger RNA cannot be directly related to the expression of protein levels or its role in pathogenesis, so we should pay more attention to the gene level as the target of HCC treatment or prognosis judgment. However, the lack of experimental evidence through bioinformatics research shows that future validation experiments are necessary to test these findings in a more formal and systematic analysis.

## 4. Conclusions

The mechanism of aflatoxin inducing HCC is not clear. Through the joint analysis of GSE127791 and GSE64041, we screened out the hub genes. With the further analysis of the hub genes, we speculated that the expression of HRG and PCK2 might be involved in aflatoxin-induced hepatocyte injury or transformation, and that the occurrence and development of HCC might be defined by the appearance of one of those key factors that would ultimately affect HCC prognosis.

## 5. Materials and Methods 

### 5.1. Screening of Datasets

The GEO database is an open high-throughput sequencing gene expression database [40]. Database screening criteria included the following: 1. Must be aligned to the human species rather than other mammalian or animal species, and 2. Discard duplicate samples that are below 3 times. 

We used "Aflatoxin Liver" as the keyword to screen the dataset GSE127791 of aflatoxin-treated primary human hepatocytes. Using "hepatocellular carcinoma" as the keyword, the sequencing datasets of normal human tissues and HCC tissues were screened. Due to the large sample size of GSE64041, it was selected as the analyzed data source. Dataset information is described in Table 1.

The experimental processing of GSE127791 datasets: induced pluripotent stem cell (iPSC)-derived human hepatocytes (on the 4th day after seeding) were treated with 0.2 µM AFB1 or AFB2, control group was treated with DMSO or deionized water the same volume for 7 consecutive days. AFB1 and AFB2 (purity ≥ 98.0%) were obtained from Sigma-Aldrich, USA. The experimental tissues of GSE64041 datasets: 60 biopsy pairs from hepatocellular carcinoma patients with unknown cause, 5 normal liver biopsies.

### 5.2. Screening of DEGs 

The dataset was analyzed by GEO 2R program in limma package in R language. Fold change (FC) and P-value are calculated by GEOR2. Benjamin & Hochberg (Error Detection Rate) was used to adjust the FC and P-value in the selection of GEO2R. 

First, we use “*p* < 0.01, |log2FC| ≥1” as the condition to analyze the effective DEGs in the dataset of GSE64041 by GEO2R, which were the DEGs between liver carcinoma and non-cancerous tissue. The same method was used to screen out the effective DEGs of GSE127791, which were the DEGs between aflatoxin-treated hepatocytes and controls. Secondly, we further identified common DEGs of two datasets which were sorted out and the common DEGs of the two datasets were analyzed by the Venn diagram web (http://bioinformatics.psb.ugent.be/webtools/Venn/). Venn diagram was used to produce overlapping results of DEGs as a graphical output.

### 5.3. Protein-Protein Interaction (PPI) Analysis

String 11.0 database is a database for calculating physical interactions and predicting protein (gene) relationships by collecting gene regulatory relationships, protein interaction relationships, protein co-expression, etc. It can build a gene (protein) interaction network by scoring the relationships between different genes (proteins) [43]. We used String to analyze protein interaction between DEGs, and used Cytoscape 3.6.1 software to visualize the protein interaction network [44,45]. Subsequently, the most significant modules in PPI network were screened by Cytosscape application MCODE. The screening criteria were: Degree cutoff = 2, node score cutoff = 0.2, K-core = 2, Max. Depth = 100.

### 5.4. KEGG Pathway and GO Term Enrichment Analysis

Gene Ontology (GO) is a comprehensive resource of computable knowledge about the functions of genes and their gene products [26]. GO describes three functions: molecular function, cellular components, and biological processes [46]. KEGG (Kyoto Encyclopedia of Genes and Genomes) is an encyclopedia of genes and genomes for biological interpretation of genomic sequences and other high-throughput data [47]. DAVID is an online functional annotation and enrichment tool (https://david.ncifcrf.gov/) [48]. To analyze the function of DEGS, we used DAVID for biological analyses. GO terms and KEGG pathways with a P-value <0.01 were considered statistically significant.

### 5.5. Screening and Analysis of Hub Genes

According to our analysis, we chose a degree ≥ 9 as the hub genes. The University of California Santa Cruz (UCSC) Genome Bioinformatics website was used to construct hierarchical clustering of hub genes (https://xena.ucsc.edu) [49]. In addition, the cBioPortal Cancer Genomics provides a Web resource for visualizing, exploring and analyzing multi-dimensional cancer genome data. The cBioPortal Cancer Genomics resource was also used to map the network of hub genes (http:/cBioPortal) [50]. The Kaplan-Meier Plotter was used to analyze the survival of hub genes (www.kmplot.com), which was a public database of mRNA expression profiles for five types of cancer (breast, ovarian, lung, gastric, and liver), from which information on gene expression and disease prognosis can be obtained. The KM Plot sources for the databases include GEO, EGA, and TCGA [51,52]. The survival curve is determined as the database evaluates the most accurate diagnosis, the most safe and effective treatment and the prognosis of the disease by combining the clinical practice and experience of the clinician with the objective scientific research evidence. The Oncomine online database was used to analyze hub gene expression in tumors and normal tissues (https://www.oncomine.org).

## Figures and Tables

**Table 1:** The information of datasets.

Series	Control Group (n)	Pathological Group (n)	Samples (Human)	Platform	Author Ref
GSE127791	6	6	iPSC-derived Hepatocytes	GPL20844	Tryndyak V, et al [41]
GSE64041	60	60	HCC with unknown cause	GPL6244	Makowska Z, et al [42]

## Supplementary Materials

The following are available online at https://www.mdpi.com/2072-6651/12/3/203/s1, Table S1: List of DEGs of two databases. Table S2: Analysis of Protein-protein interaction. Table S3: The first 30 common DEGs Table. S4: Enrichment Analysis.
