# Supplementary material for: Molecular Mechanism of Aflatoxin-Induced Hepatocellular Carcinoma Derived from a Bioinformatics Analysis
Source: Toxins (Basel). 2020 Mar 23;12(3):203. doi: 10.3390/toxins12030203 (PMC7150856; doi:10.3390/toxins12030203)
Supplement: Supplementary file 1 [file toxins-12-00203-s001.pdf]

Supplementary Materials: Molecular Mechanism of Aflatoxin-Induced Hepatocellular Carcinoma Derived from a Bioinformatics Analysis

Peirong Cai, Hao Zheng, Jinjin She, Nannan Feng, Hui Zou, Jianhong Gu, Yan Yuan, Xuezhong Liu, Zongping Liu and Jianchun Bian

Table S1. List of DEGs of two database.

Gene	Group	logFC
HIST1H2BJ	up	0.216786
TTC13	up	0.500696
CCNA2	up	1.212165
C17orf62	up	0.133877
TP53I3	up	0.694476
DIABLO	up	0.144145
HSD17B14	up	0.430577
MAP4	up	0.224261
MPPED2	up	0.42813
ZNF648	up	0.773388
RUNX1	up	0.238824
CDC42SE1	up	0.228416
SFN	up	0.474267
RRM2B	up	0.265833
TOB2P1	up	0.388853
PPP4R4	up	0.341626
DNMT3B	up	0.119307
SULT1C2	up	0.95617
SLC7A11	up	1.951166
PSEN2	up	0.188816
SRD5A3	up	0.23392
TMEM97	up	0.297854
PPM1D	up	0.180746
ADAMTS18	up	0.162022
MAPT	up	0.369837
CYB561D2	up	0.135514
NOX4	up	0.569495
SNORA38	up	0.307846
MTBP	up	0.548337
CTNNA2	up	0.325341
REEP6	down	−0.55495
ZG16	down	−1.11962
ACSM3	down	−1.31213
ALDOC	down	−0.59397
GBP7	down	−0.80788
ASS1	down	−0.87842
F7	down	−0.48586
CYP4A22	down	−1.4601
KLKB1	down	−1.12874
SEBOX	down	−0.20308
NUGGC	down	−0.39501
ABCG8	down	−0.47544

PHGDH	down	−1.06515
GCGR	down	−0.90499
LOC100506990	down	−0.56405
GIMAP6	down	−0.25108
KANK4	down	−0.24153
SPTLC3	down	−0.62646
PHYH	down	−0.61999
ADCY1	down	−0.26611
SLC23A1	down	−0.34512
SLC10A1	down	−1.01943
SLC6A1	down	−0.53629
ABCA6	down	−0.47112
AKR1D1	down	−1.72156
INHBE	down	−1.02093
SLC6A12	down	−0.64028
CRYL1	down	−0.4662
OTC	down	−0.68225
LY9	down	−0.24853
RTP3	down	−0.50277
KHK	down	−0.64067
P4HTM	down	−0.17575
AMPD1	down	−0.75087
AFM	down	−1.16342
MST1	down	−0.76516
SMIM24	down	−0.87228
PLCG2	down	−0.37574
ACSM2A	down	−0.62364
ADH1A	down	−0.75964
IYD	down	−0.64891
MYO7A	down	−0.41334
GBA3	down	−1.6779
NR1I2	down	−0.61894
FTCD	down	−0.85165
ANKRD55	down	−0.47589
AGMAT	down	−0.45476
GATM	down	−0.28269
SLC51A	down	−0.76266
C6	down	−0.86971
CDK10	down	−0.16098
CACNA2D1	down	−0.34166
HRG	down	−0.85216
PCOLCE	down	−0.56773
BHMT	down	−1.14602
PCK2	down	−0.65342
DAO	down	−0.8701
SLED1	down	−0.65805
SLC38A4	down	−1.06574
LINC01558	down	−0.51472
ACSF2	down	−0.2716
ADH1C	down	−0.88209
CA5A	down	−0.43549
LINC00341	down	−0.2783
MCTP2	down	−0.67089
TMEM178A	down	−0.28422

SAMD5	down	−0.29534
SLC22A7	down	−1.02521
FBP1	down	−1.27167
ACACB	down	−0.9721
AGXT	down	−0.77553
IFITM1	down	−0.43919
COMT	down	−0.46629
C9	down	−1.67921
LDHD	down	−0.60123
UBA7	down	−0.20893
TNFSF10	down	−0.51718
HAO1	down	−0.60427
LCN12	down	−0.20262
RAB26	down	−0.59448
CHST13	down	−0.20007
CFHR4	down	−0.79389
S100A14	down	−0.47215
ARG1	down	−0.85735
GALM	down	−0.37868
CTH	down	−0.96883
ATF5	down	−0.82007
EPHX2	down	−1.04077
HPD	down	−0.54495
ECM2	down	−0.27385
ALDH8A1	down	−1.32975
GNMT	down	−1.53905
ADK	down	−0.53786
GLYAT	down	−0.95036
ABCG5	down	−0.46117
CLYBL	down	−0.6061
OAF	down	−0.45157
SARDH	down	−0.75833
ADH6	down	−0.81729
ALDOB	down	−0.6979
SLCO4C1	down	−2.26324
GALNT16	down	−0.31728

Table S2. Analysis of Protein protein interaction.

#Node1	Node2	Combined_Score
ASS1	OTC	0.999
ABCG8	ABCG5	0.992
HAO1	AGXT	0.99
ARG1	OTC	0.976
AMPD1	ADK	0.97
FBP1	ALDOB	0.967
HAO1	DAO	0.961
CTH	BHMT	0.961
FBP1	ALDOC	0.958
AGXT	DAO	0.957
ALDOB	KHK	0.957
EPHX2	AGXT	0.953
GATM	ARG1	0.948
SARDH	DAO	0.944

GCGR	ADCY1	0.933
GNMT	AGXT	0.932
SRD5A3	AKR1D1	0.93
KHK	ALDOC	0.93
ADH6	COMT	0.93
C6	C9	0.928
EPHX2	HAO1	0.928
GATM	GNMT	0.926
GATM	AGXT	0.926
GATM	SARDH	0.924
EPHX2	DAO	0.924
SARDH	AGXT	0.919
HIST1H2BJ	DNMT3B	0.918
ADH1C	ADH1A	0.916
GATM	DAO	0.91
HIST1H2BJ	CCNA2	0.909
LDHD	OTC	0.908
GNMT	DAO	0.907
GNMT	SARDH	0.902
MST1	SFN	0.9
HIST1H2BJ	RUNX1	0.9
ASS1	FTCD	0.873
HAO1	C9	0.863
GNMT	BHMT	0.827
SARDH	FTCD	0.822
ALDOB	ALDOC	0.806
SPTLC3	AGXT	0.805
GNMT	FTCD	0.791
ASS1	ARG1	0.785
PHGDH	AGXT	0.77
AGXT	FTCD	0.766
PSEN2	MAPT	0.765
ADH1C	COMT	0.756
COMT	ADH1A	0.749
AGXT	HRG	0.726
FTCD	C9	0.723
ASS1	PHGDH	0.722
SARDH	BHMT	0.715
AGMAT	ASS1	0.698
FBP1	PCK2	0.697
SLC22A7	SLC10A1	0.695
ABCG5	SLC10A1	0.689
KLKB1	HRG	0.684
HRG	AFM	0.683
AGXT	ACSM3	0.678
NR1I2	SLC10A1	0.668
DIABLO	TNFSF10	0.662
HAO1	FTCD	0.66
AGMAT	ACACB	0.657
AGXT	ALDH8A1	0.657
FTCD	HRG	0.656
ALDH8A1	HRG	0.655
SULT1C2	FTCD	0.634
SULT1C2	AGXT	0.628

DIABLO	GALM	0.626
SLC51A	SLC10A1	0.612
ABCG8	SLC10A1	0.61
AGMAT	AGXT	0.61
CTH	DAO	0.607
GNMT	CTH	0.606
SULT1C2	ALDH8A1	0.595
HAO1	LDHD	0.594
ACSM3	ALDH8A1	0.594
FTCD	ALDH8A1	0.589
ASS1	ADK	0.586
GATM	ASS1	0.579
F7	KLKB1	0.579
FTCD	ACSM3	0.576
ALDOB	SULT1C2	0.576
SLC6A1	DAO	0.569
SLC22A7	SLCO4C1	0.567
F7	FTCD	0.566
ASS1	CTH	0.556
KLKB1	C6	0.55
F7	C6	0.548
FBP1	AGXT	0.543
CTH	PHGDH	0.542
ACACB	AGXT	0.54
NR1I2	ABCG5	0.539
ACSF2	ACACB	0.538
SARDH	ACSM3	0.536
NR1I2	ABCG8	0.535
C6	HRG	0.534
ACSM3	HRG	0.533
ALDOB	OTC	0.529
HPD	BHMT	0.529
SULT1C2	SLC51A	0.523
HRG	IYD	0.522
FTCD	C6	0.522
SLC51A	HRG	0.522
AGXT	SLC51A	0.519
SLC51A	FTCD	0.512
SLC51A	ALDH8A1	0.512
SULT1C2	ACSM3	0.512
ALDH8A1	C6	0.511
SLC51A	ACSM3	0.509
AGMAT	OTC	0.508
GATM	AGMAT	0.504
AGXT	PCK2	0.503
FBP1	BHMT	0.502
SARDH	CTH	0.5
FTCD	IYD	0.499
GALM	ALDOC	0.498
CRYL1	PHYH	0.494
SLC22A7	ABCG8	0.493
ADK	OTC	0.478
AGMAT	PHGDH	0.476
FTCD	HPD	0.476

RRM2B	TP53I3	0.475
SLC22A7	ABCG5	0.471
ALDOB	GALM	0.47
ATF5	SLC7A11	0.47
CTH	SLC7A11	0.468
SLC6A12	GATM	0.465
SLC22A7	NR1I2	0.462
C6	IYD	0.461
ALDH8A1	KLKB1	0.459
FTCD	KLKB1	0.459
GATM	OTC	0.457
FBP1	HPD	0.456
CTH	OTC	0.456
ACSM3	PCK2	0.455
FBP1	GNMT	0.454
GCGR	PCK2	0.453
EPHX2	CYP4A22	0.453
PPM1D	RRM2B	0.45
COMT	DAO	0.45
KLKB1	C9	0.449
HAO1	ALDH8A1	0.443
PHGDH	SLC7A11	0.442
AKR1D1	SLC10A1	0.436
ADH1C	ALDOB	0.436
SLC51A	AKR1D1	0.436
ALDOB	PCK2	0.435
CTH	NOX4	0.433
FBP1	LDHD	0.432
LDHD	PCK2	0.429
FTCD	PCK2	0.427
PCK2	OTC	0.426
CLYBL	AGXT	0.426
ADK	GALM	0.425
AGXT	HPD	0.422
FTCD	BHMT	0.421
NR1I2	SLCO4C1	0.419
AGMAT	FTCD	0.416
ACACB	PCK2	0.414
NR1I2	SLC51A	0.413
F7	OTC	0.411
ADH6	ALDH8A1	0.407
ASS1	BHMT	0.407
CYP4A22	NR1I2	0.405
SLC6A12	BHMT	0.404
MST1	KLKB1	0.4

Table 3. The first 30 common DEGs.

Gene Symbol	*p*-value
ADK	$2.39E^{-08}$
ACACB	$5.47E^{-13}$
ABCG8	0.000599
ABCG5	0.00149
SLC22A7	0.00000161

PHGDH	2.06E^{-09}
SULT1C2	0.000378
SLC10A1	0.00000629
NR1I2	0.000000301
KLKB1	2.85E^{-10}
HAO1	0.00148
C6	4.45E-08
AGMAT	0.0000225
SARDH	2E^{-10}
GNMT	4.31E^{-10}
FBP1	9.28E^{-17}
BHMT	3.06E^{-08}
ALDOB	0.0000876
ACSM3	2.25E^{-12}
SLC51A	0.00001
PCK2	0.000000592
HRG	0.000588
GATM	0.00146
DAO	0.00000212
CTH	0.00000475
ASS1	5.1E-13
OTC	0.00019
ALDH8A1	4.77E^{-12}
FTCD	4.09E^{-10}
AGXT	0.000000308

Table 4. Enrichment Analysis.

Category	Term	Count	*p*-value
KEGG_PATHWAY	hsa01100:Metabolic pathways	36	8.64E-13
KEGG_PATHWAY	hsa01130:Biosynthesis of antibiotics	14	1.81E-08
KEGG_PATHWAY	hsa00260:Glycine, serine and threonine metabolism	8	2.94E-08
KEGG_PATHWAY	hsa00010:Glycolysis / Gluconeogenesis	8	1.38E-06
KEGG_PATHWAY	hsa01230:Biosynthesis of amino acids	7	3.10E-05
KEGG_PATHWAY	hsa00350:Tyrosine metabolism	5	2.10E-04
KEGG_PATHWAY	hsa04976:Bile secretion	6	2.89E-04
KEGG_PATHWAY	hsa00051:Fructose and mannose metabolism	4	0.002491207
KEGG_PATHWAY	hsa01200:Carbon metabolism	6	0.002705011
KEGG_PATHWAY	hsa04146:Peroxisome	5	0.005419719
KEGG_PATHWAY	hsa00330:Arginine and proline metabolism	4	0.008827609
GOTERM_BP_DIRECT	GO:0006000~fructose metabolic process	4	1.68E-05
GOTERM_BP_DIRECT	GO:0046487~glyoxylate metabolic process	4	3.43E-04
GOTERM_BP_DIRECT	GO:0030388~fructose 1,6-bisphosphate metabolic process	3	5.73E-04
GOTERM_BP_DIRECT	GO:0010043~response to zinc ion	4	9.05E-04
GOTERM_BP_DIRECT	GO:0055114~oxidation-reduction process	11	0.001143987
GOTERM_BP_DIRECT	GO:0042493~response to drug	8	0.00115403
GOTERM_BP_DIRECT	GO:0071377~cellular response to glucagon stimulus	4	0.001233969
GOTERM_BP_DIRECT	GO:0006094~gluconeogenesis	4	0.001629052
GOTERM_BP_DIRECT	GO:0006069~ethanol oxidation	3	0.00177107
GOTERM_BP_DIRECT	GO:0000050~urea cycle	3	0.00177107
GOTERM_BP_DIRECT	GO:0051289~protein homotetramerization	4	0.003960316
GOTERM_BP_DIRECT	GO:0042632~cholesterol homeostasis	4	0.004749078
GOTERM_BP_DIRECT	GO:0007584~response to nutrient	4	0.007113888

GOTERM_BP_DIRECT	GO:0055085~transmembrane transport	6	0.00960036
GOTERM_CC_DIRECT	GO:0070062~extracellular exosome	39	5.97E-09
GOTERM_CC_DIRECT	GO:0005759~mitochondrial matrix	9	3.09E-04
GOTERM_CC_DIRECT	GO:0005782~peroxisomal matrix	4	0.001770974
GOTERM_CC_DIRECT	GO:0005777~peroxisome	5	0.002140402
GOTERM_CC_DIRECT	GO:0005829~cytosol	29	0.004861998
GOTERM_MF_DIRECT	GO:0004024~alcohol dehydrogenase activity, zinc-dependent	3	4.07E-04
GOTERM_MF_DIRECT	GO:0003824~catalytic activity	7	4.76E-04
GOTERM_MF_DIRECT	GO:0004022~alcohol dehydrogenase (NAD) activity	3	5.67E-04
GOTERM_MF_DIRECT	GO:0003996~acyl-CoA ligase activity	3	7.54E-04
GOTERM_MF_DIRECT	GO:0016597~amino acid binding	3	0.005928252
